# Osteolytic lesion in polycythemia vera: First report and review of literature

**DOI:** 10.1002/jha2.420

**Published:** 2022-03-27

**Authors:** Verna Cheung, James England, Dawn Maze, Hassan Sibai

**Affiliations:** ^1^ Princess Margaret Cancer Centre University of Health Network Toronto Canada; ^2^ Faculty of Nursing University of Toronto Toronto Ontario Canada; ^3^ Department of Medicine University of Toronto Toronto Ontario Canada

**Keywords:** bone pain, myeloproliferative neoplasms (MPNs), osteolytic lesion, polycythemia vera

## 1 INTRODUCTION

Myeloproliferative neoplasms (MPNs) are a group of rare clonal disorders of hematopoietic progenitor cells that are associated with morbidity from disease‐related symptoms, thrombotic events, and risk of transformation to acute myeloid leukemia (AML) [1]. The three most common MPNs are polycythemia vera (PV), essential thrombocytosis (ET), and myelofibrosis (MF). Patients with MPN can experience a constellation of debilitating symptoms that negatively impact their quality of life [2, 3, 4, 5]. In prior surveys of patients with MPNs, bone pain was the fourth most common symptom reported by 44% of patients and rated as “very severe” in up to a third of MF patients [2, 3, 4, 5].

In very rare situations, patients with MPN can develop osteolytic lesions [6, 7, 8, 9, 10, 11, 12, 13, 14, 15, 16, 17, 18, 19, 20, 21]. We report the first case of an osteolytic lesion in a chronic‐phase PV patient, and review published case reports of MPN patients with osteolytic lesions to summarize the clinical characteristics and implications for patient care.

## 2 PRINCESS MARGARET DATA

The MPN program at the Princess Margaret Cancer Centre is the largest MPN program in Canada. The MPN program has surveyed 1096 MPN patients regarding their symptoms, 208 MPN patients reported experiencing bone pain. Among these 208 patients, 17% have PV, 15% have ET, and 50% have MF (which includes post PV/ET‐MF, prefibrotic‐MF or PMF). Of 208 patients, 76 (37%) reported moderate or higher levels of bone pain (5/10 or greater based on the MPN‐symptoms survey).

### 2.1 Case Report

**FIGURE 1:**
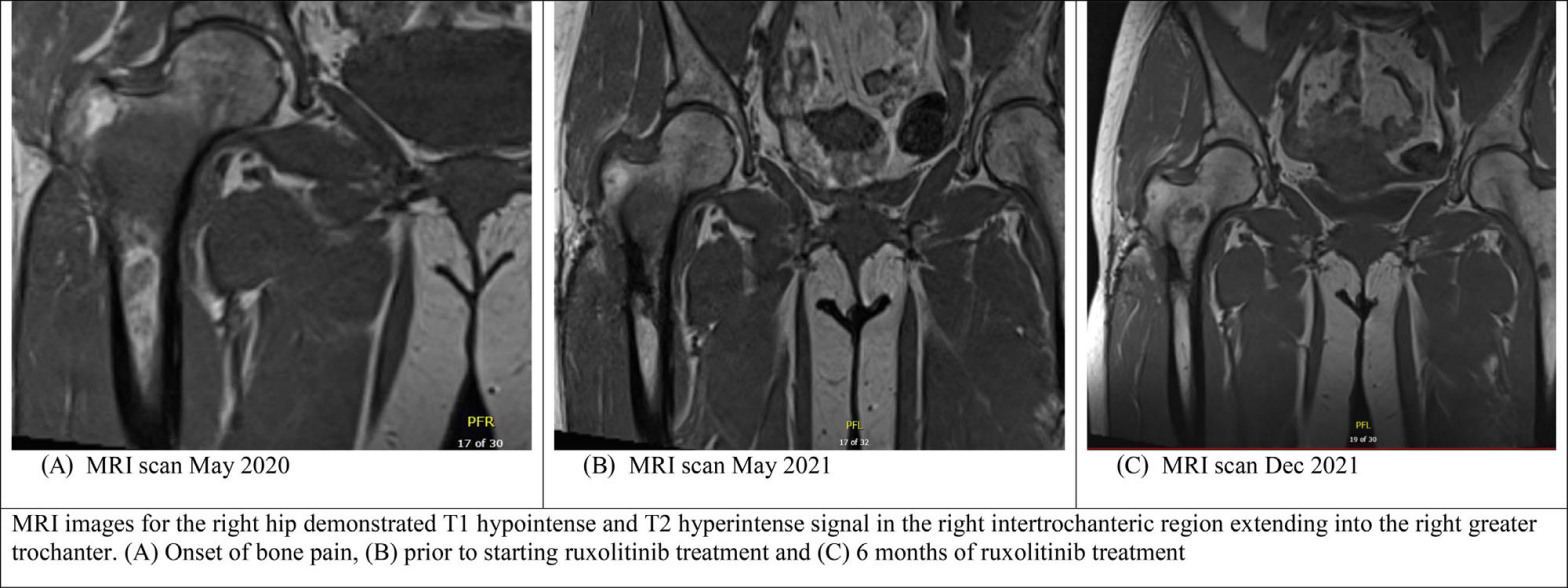
Magnetic resonance imaging (MRI) scans of the right femur

In 2019, a 59‐year‐old woman was found on routine bloodwork to have elevated hemoglobin (185 g/L) and hematocrit (0.58). All other counts were within normal limits. Molecular testing confirmed *JAK2* V617F mutation, erythropoietin level was < 2 IU/L, and bone marrow biopsy demonstrated a hypercellular marrow with increased erythropoiesis and granulopoiesis, establishing a diagnosis of PV. The patient was started on aspirin 81 mg daily with phlebotomies as needed. In 2020, she developed excruciating right hip pain, leading to multiple emergency department presentations. Magnetic resonance imaging (MRI) of right hip demonstrated T1 hypointense and T2 hyperintense signal in the right intertrochanteric region extending into the right greater trochanter measuring 3.9 × 3.5 × 2.6 cm (Figure 1A). Computed tomography (CT) scan confirmed a lytic lesion within the right proximal femur. Investigations for other malignancies including multiple myeloma were normal. ^18^Fluorodeoxyglucose positron emission tomography (PET) scan confirmed the right femur lesion with increased metabolic activity, but found no other abnormalities. Subsequent CT‐guided biopsy of the right proximal femur reported hematopoietic tissue with hypercellularity and panmyelosis consistent with PV.

Coinciding with the osteolytic lesion the patient lost phlebotomy requirements (hemoglobin 125 g/L, hematocrit 0.40). A repeat bone marrow biopsy had no significant changes from prior assessment (Table S1). She reported no constitutional symptoms, but impaired mobility related to right hip pain. Ruxolitinib was initiated at 5 mg BID as a trial for pain control based on prior case reports [2, 18]. After 6 months of therapy, the hip pain improved on patient self‐assessment from 10/10 to 3/10, blood counts remained stable, and follow‐up MRI demonstrated decrease in edematous fluid surrounding the lesion (Figure 1C).

## 3 LITERATURE REVIEW

We conducted an English literature search using Google scholar, PubMed, and Medline, for studies, reviews, case series, and case reports of patients with diagnosis of PV, ET, PMF, and MPN‐unclassified associated with osteolytic bone lesion from 1970 to July 2021.

In total, 16 case reports were found, highlighting that osteolytic lesion secondary to MPNs is rare (Table 1) [6, 7, 8, 9, 10, 11, 12, 13, 14, 15, 16, 17, 18, 19, 20, 21]. All patients reported new or worsening bone pain as a presenting symptom. Osteolytic lesions were solitary in five patients; while 11 had multiple concurrent lesions. In 15 of 16 cases, patients had an established diagnosis of MF; PMF in 7 and secondary MF in 8 patients. One patient with an established PV diagnosis presented with osteolytic lesion concurrently with progression to secondary AML [17]. Median age of diagnosis was 62.5 (range 30–83 years of age), with nine female and seven male patients. The time between diagnosis and the onset of an osteolytic lesion ranged from immediately to 33 years. Symptom burden was significant with patients requiring multiple hospital admissions and interventions following identification of osteolytic lesions. Treatments provided for bone pain included palliative radiation [8, 20], orthopedic surgery for pathological fracture [11, 19], steroid therapy [6, 16], JAK‐inhibitor [18], and chemotherapy [6, 10, 14]. In 14 cases, therapies only provided temporary symptomatic relief; and the osteolytic lesions either remained unchanged [6, 7, 8, 9, 10, 11, 13, 15, 16, 17, 19, 21] or additional osteolytic lesions developed [20].

**TABLE 1:** Clinical cases reports for osteolytic lesion in myeloproliferative neoplasm (MPN)

Articles	Patient age, gender, and diagnosis	History and presentation	Site of osteolytic lesion	Diagnostic work‐up	Treatment	Response	Outcomes
Licht et al., 1973 [6]	40F CML to MF	In 1966, incidental finding of leukocytosis and thrombocytosis, no symptoms, no splenomegaly Bone marrow biopsy confirmed CML In April 1971 developed severe bone pain, found to have left shift and leukopenia	Femur, ribs, pelvis, and skull	Repeat bone marrow confirmed myelofibrosis	Prednisone and 6‐mecaptopurine Patient admitted as condition continue to decline	N/A	Death: 1 month from initial finding of lytic lesion (May 1971)
Rudders & Kilcoyne 1974 [7]	73F MPN to MF	In December 1966, found to have leukocytosis and thrombocytosis Bone marrow biopsy suggestive of MPN In July 1971, presented with chest pain, anemia, and weakness,	Ribs, pelvis, femur, and skull	Repeat marrow showed myelofibrosis	No treatment interventions	N/A	Death: 17 months from initial finding of osteolytic lesion (Dec 1972)
Leimert et al., 1978 [8]	49M Post PV MF	Diagnosed in 1971 with PV, treated with phlebotomies In September 1976 developed persistent knee pain and constitutional symptoms	Fibula, tibias, pelvis, and lumbar spine	Bone marrow biopsy revealed fibrosis in marrow	Radiation therapy to knees	No improvement	Death: 4 months from initial finding of osteolytic lesion (Jan 1977)
Kosmidis et al., 1980 [9]	52 F Post PV‐MF	In 1959 initially diagnosed with PV based on elevated blood counts In January 1976 disease progressed to MF, with symptomatic splenomegaly, anemia In February 1978 severe acute chest pain	Ribs and long bone	Bone biopsy of osteolytic lesion (no further details provided) confirmed extensive fibrosis	Analgesics and transfusion support	N/A	Death: 5 months from initial finding of osteolytic lesion (July 1978)
Gruber & Osby, 1987 [10]	64F PMF	Diagnosed with PMF in November 1974 In 1979 developed back pain that progressively worsened over the years	Skull, scapulae, humeri and vertebrae	Bone biopsy (site not specified) confirmed hematopoietic cells Myeloma work‐up negative	Analgesics, busulphan, and transfusion support	Temporary relief	Death: 9 years 11 months from initial finding of osteolytic lesion (October 1984)
Clutterback et al., 1995 [11]	59M PMF	33‐year history of MF, stable disease Patient presented with pneumonia, weakness, and paresthesia in his left leg with flaccid paralysis of the left foot	Left femur	Bone biopsy of left femur revealed hematopoietic cells, no evidence of other malignancy	Intramedullary nailing of the femur	Recovered from the surgery and discharged	Death due to septicemia after 6 months
Sadoun et al., 1997 [12]	30M Hyper‐eosinophilic syndrome transform to MF	In 1989, diagnosed with hypereosinophilic syndrome, confirmed by bone marrow aspirate In July 1992 admitted with severe hypercalcemia (3.7 mmol/l), anemia, bone pain	Pelvis	Repeat bone marrow biopsy confirmed transformation to MF cytogenetics remained normal	Oct 1992 underwent bone marrow transplant	12‐month post‐transplant repeat pelvic x‐ray showed resolution of osteolytic lesion	Alive at last follow‐up
Sideris et al., 2006 [13]	72M Post PV MF	Presented to hospital with low back pain and anemia	Rib, sternum, vertebra, pelvis and calcaneus	Bone biopsy (vertebrae) most aligned with MPN,	N/A	N/A	Death: 11 months after progression to AML
Jurisic et al., 2008 [14]	49F PMF to AML	In 1991 presented with abdominal pain, splenomegaly, anemia, and general malaise Bone marrow biopsy confirmed diagnosis of PMF Condition continued to deteriorate and developed bone pain.	Pelvis and long bones	Multiple myeloma work‐up negative Bone marrow biopsy showed 72% blast indicating transformation to AML	Cytosine‐arabinoside, did not achieve remission Subsequently treated with hydroxyurea did not achieve remission	Did not achieve remission	Alive at last follow‐up Transfusion support
Merry & Aronowitz. 2010 [15]	83M PMF	7‐year history of MF diagnosis, managed with transfusion support and thalidomide Worsening fatigue and weakness, leading to fall at home	Left wrist, hand, and forearm	N/A	Cast to left arm and wrist Transfusion support	N/A	Death: 1 month after admission (cause unclear)
Sacre et al, 2010 [16]	44F PMF	Longstanding history of systemic lupus erythematosus In 2002, low hemoglobin and platelets. Bone marrow biopsy confirmed diagnosis of MF In 2005, developed bilateral shoulder pain	Bilateral shoulders and humerus	Patient declined bone biopsy of osteolytic lesion	Low dose prednisone and hydroxychloroquine	Osteolytic lesion unchanged	Alive at time of publication in 2010
Chambers et al., 2016 [17]	62F PV transform to AML	Presented to hospital with increasing weakness, confusion, and also suffered a fall	Lateral aspect of T2 vertebrae and skull	Bone marrow biopsy confirmed PV transform to AML Myeloma work‐up negative	Palliative care	Admitted to palliative unit	Unclear survival duration
Bucelli et al., 2018 [18]	59F PMF	26‐year history of PMF Presented with left upper arm pain	Left humeral shaft	Bone biopsy of lytic lesion confirmed grade 3 MF	Ruxolitinib 15 mg BID	After 9 months of ruxolitinib therapy, resolution of osteolytic lesion	Alive at last follow‐up
Duval et al., 2019 [19]	82M JAK2+ ET transform to PV to post PV‐MF	In 1994, diagnosed with ET transform to PV In 2015 due to anemia and splenomegaly repeat marrow showed post PV MF (MF grade 3) In July 2016, admitted due to decline in general health, and also developed right shoulder pain	Entire axial skeleton	Bone biopsy of an osteolytic lesion (no further specification) ‐ grade 3 MF Bone marrow biopsy confirmed AML	Orthopedic surgical procedure to prevent fracturing	Transferred to palliative care unit	Death: 2 months after admission to palliative care unit
Burnham et al., 2020 [20]	63M PMF JAK2+	3‐year history of PMF Underwent stem cell transplant Relapsed 1 year post‐stem cell transplant Presented with lateral left hip and thigh pain	Proximal left femur, then right femur, shoulder, and calcaneus	Bone biopsy of femoral lesion confirmed PMF	Radiation therapy, 25 Gy in 10 Fraction	Temporary pain improvement, further development of lytic lesions	Death: 10 months from initial osteolytic lesion presentation
Johnson & Alkhateeb, 2021 [21]	58F Secondary MF CALR type 1	Long‐standing history of MPN diagnosis, with subsequent diagnosis of stage IIIA Merkel cell carcinoma Developed severe hip pain 1 year after Merkel cell carcinoma	Pelvis, femur, and scapula	Bone biopsy (pelvic) confirmed MPN with fibrosis No evidence of Merkel cell carcinoma cytogenetics: del 5q and 17p	Underwent stem cell transplant	Disease relapse to MF	Death: 94 days post‐transplant

Abbreviations: AML, acute myeloid leukemia; CML, chronic myelogenous leukemia; ET, essential thrombocytosis; MF, myelofibrosis; MPN, myeloproliferative neoplasm; N/A, not applicable; PMF, prefibrotic myelofibrosis; PV, polycythemia vera.

In all 16 cases investigations revealed progressive disease (i.e., worsening fibrosis, accelerated phase MPN or AML) along with the presence of osteolytic lesions; though diagnostic procedures were often delayed until further symptoms or cytopenias developed [6, 7, 8, 9, 10, 11, 12, 13, 14, 15, 16, 18, 19, 20, 21]. Chambers et al. reported a patient with longstanding history of PV managed with hydroxyurea who presented to her local hospital after a fall and imaging revealed the presence of an osteolytic lesion [17]. A bone marrow biopsy performed to investigate new anemia demonstrated transformation from PV to AML [17]. Disease progression or death was observed within 12 months of identification of osteolytic lesion in nine cases [6, 7, 8, 9, 11, 13, 15, 19, 20].

## 4 DISCUSSION

### 4.1 Development of osteolytic lesions

The etiology of osteolytic lesions in MPN is not fully understood. Activation of the JAK‐STAT pathway leads to altered hematopoiesis and proliferation of one or more cell lines, associated with modifications to the microenvironment of the bone marrow [18, 19, 20, 22, 23]. Microenvironment changes in the bone marrow include abnormal production of granulocytes, megakaryocytes, fibroblasts, osteoblasts, and release of pro‐inflammatory cytokines, which lead to marrow fibrosis, osteosclerosis, compressive atrophy, and bone destruction [18, 22, 23, 24] Osteosclerosis can be seen in 40%–70% of patients with MF, and less commonly in other MPNs [18, 23, 24]. The literature suggests that osteolytic lesions occur in areas where there has been significant osteosclerosis and bone destruction [18, 23, 24]. Another possible cause for osteolytic lesions may be as a result of leukemic transformation of MPN from leukemic bone infiltration or focal bone destruction by tumor necrosis factor‐alfa released by leukemic cells [14, 22, 24].

### 4.2 Clinical significance of osteolytic lesions in MPN

The occurrence of osteolytic lesion in MPN patients, independent of another malignant process, is rare. The summarized experience from the literature raises the concern that osteolytic lesions are a sign of disease progression and denote a poorer prognosis [6, 7, 8, 9, 10, 11, 12, 13, 14, 15, 16, 17, 18, 19, 20, 21]. Although high‐risk mutations and/or unfavorable cytogenetics can also drive disease progression in MPN [1, 23], extended molecular mutation were not provided in the 16 case reports. One case report did describe a patient with unfavorable cytogenetics [7].

Reported medical and surgical therapies have demonstrated some temporary symptomatic benefit, though most patients have persistent or new lesions. Radiographic resolution of osteolytic lesions has only been reported in two of the 16 cases. One case had resolution following allogeneic stem cell transplant [12]. Bucelli et al. reported a patient with MF and osteolytic lesions to her left proximal humerus in whom ruxolitinib was started for symptomatic splenomegaly. After 9 months of treatment, spleen size was decreased and bone pain was improved; reimaging confirmed resolution of the osteolytic lesion [18]. This suggests ruxolitinib might have altered the microenvironment of the bone marrow leading to resolution of the osteolytic lesions, but further research is needed to explore this possibility [18, 25, 26, 27]. In our presented case, ruxolitinib has led to improved pain control and improvement on MRI after limited follow‐up.

Based on the 16 case reports, and our institute's experience, the occurrence of osteolytic lesion in MPN patients is rare. However, the occurrence of osteolytic lesions is associated with significant bone pain, declined quality of life, aggressively behaving disease, an increased risk of disease progression or leukemic transformation, and limited survival [6, 7, 8, 9, 10, 11, 12, 13, 14, 15, 16, 17, 18, 19, 20, 21].

### 4.3 Implications for patient care

The presence of persistent worsening bone pain in patients with MPN should prompt imaging with CT scan followed by PET scan as needed to investigate for lytic lesions, especially with the poor prognosis associated with this finding. Given that osteolytic lesions can occur in other hematological malignancies (i.e., multiple myeloma), ruling out concurrent disease is necessary (i.e., serum protein electrophoresis, parathyroid hormone) [10, 14, 17]. If no other malignancy is found in an MPN patient, we recommend both biopsy of the osteolytic lesion to confirm etiology and repeat bone marrow biopsy to evaluate for disease progression.

## 5 CONCLUSION

We report the first case of chronic‐phase PV with osteolytic lesion with clinical response to ruxolitinib. The occurrence of osteolytic lesions in MPN is rare and characterized by excruciating bone pain, aggressively behaving disease, and may indicate an overall poor prognosis. Further investigation and attention to bone pain with MPN is warranted to reduce symptom burden and identify patients at higher risk of progression.

## FUNDING INFORMATION

Funding support for this project from the Corresponding author Hassan Sibai.

## CONFLICT OF INTEREST

Dawn Maze has research support, honoraria, advisory board, and consultancy for Novartis, Celgene/Bristol‐Myers Squibb, PharmaEssentia, Takeda, and Pfizer. The other authors have no conflict of interest to disclose.

## ETHICS STATEMENT

Written consent for the publication of a case report was obtained from the patient. In addition, Institutional Ethic Review was not required for case report by the University Health Network.

## Supporting information

Supporting Information
