# Supplementary material for: Osteolytic lesion in polycythemia vera: First report and review of literature
Source: EJHaem. 2022 Mar 27;3(2):526–32. doi: 10.1002/jha2.420 (PMC9176128; doi:10.1002/jha2.420)
Supplement: Supplementary file 1 — Supporting Information [file JHA2-3-526-s001.docx]

|  | **Initial diagnosis (Jan 2019)** | | | | | | **Repeat marrow (June 2020)** | | | | | |
| --- | --- | --- | --- | --- | --- | --- | --- | --- | --- | --- | --- | --- |
| Peripheral blood counts | Hb | HCT | MCV | plts | WBC | Neuts | Hb | HCT | MCV | plts | WBC | Neuts |
|  | 185g/L | 0.58 | 102 fL | 234x10^9^/L | 6.5x10^9^/L | 3.8x10^9^/L | 125g/L | 0.40 | 77 fL | 227x10^9^/L | 6.9 x10^9^/L | 4.3 x10^9^/L |
| EPO level | Less than 2.0 IU/L | | | | | | Not repeated | | | | | |
| LDH | 240 U/L | | | | | | 197 U/L | | | | | |
| Lipids | Cholesterol in HDL – not done  Cholesterol in LDL – not done  Triglyceride – not done | | | | | | Cholesterol in HDL – 1.49 mmol/L  Cholesterol in LDL – 4.18 mmol/L  Triglyceride – 1.0 mmol/L | | | | | |
| Blood film review | Anisocytosis – present  Macrocytes – 1+ | | | | | | Hypochromia – present  Anisocytosis – present  Microcytosis – present | | | | | |
| Next generation sequencing | JAK2 V617F allele frequency 9%  TET2 allele frequency 5% | | | | | | Not repeated | | | | | |
| Morphologic description | Cellularity 60%  Erythropoiesis relatively hypercellular, few dysplastic erythroblast are seen.  Granulopoiesis relatively hypocellular.  Megakaryopoiesis normocellular, with normal morphology. Clusters not seen.  CD34+ cells 2%.  MF1 by reticulin and trichrome staining | | | | | | Cellularity 80%  Erythropoiesis hypercellular. Subtle increase of early erythroblasts.  Granulopoiesis relatively hypercellular.  Megakaryopoiesis normocellular with normal morphology.  CD34+ cells not increased  MF 1 to 2 (in few areas) by reticulin and trichrome staining. | | | | | |
| Cytogenetics | 46XX | | | | | | 46XX | | | | | |
| Diagnosis | Most aligned with PV | | | | | | Findings consistent with previous diagnosis MPN PV | | | | | |
| Treatment | ASA 81mg and phlebotomies (last phlebotomy July 2019) | | | | | | ASA 81mg | | | | | |

**Supplementary Table 1 – Patient clinical characteristic from initial diagnosis to time of repeat marrow.**
